# Phytolocca Decandra

**Published:** 1873-11

**Authors:** C. H. Tidd


Phytolocca Decandra.—*C. H. Tidd, M.D.*—I am now trying it in a case of chronic rheumatism, giving it in 5 gr. doses ter die. And although the treatment has not been prolonged sufficiently to enable me to say positively as to the final result, yet I predict a radical cure, as the patient expresses himself as feeling better now than for two years; being enabled to walk without his cane and put on his collar, which he has not done for more than three years. I intend trying it in two cases of syphilitic neuralgia and one of secondary eruptions and shall take pleasure in reporting to you the result; although I am quite sure that I shall see good results in these cases as well as in those already cited. For internal use I think I should prefer a tincture prepared of about the strength of the following:

℞. Phytoloccæ Radicis ℥ij.

Spirit. Frumenti Oj. M.

Sig. 15 gtts. to ʒj. pro re nata; this strength giving us about grs. 5 to each drachm.

I apprehend that in the treatment of cancer, this agent is destined to come into extensive use so soon as it is thoroughly understood, and had I a case of that kind to treat to-day I should most certainly give this remedy the preference over any other that I can think of, and should not be compelled to resort to the knife, unless the disease was far advanced before I saw the case. In such cases I should use it both externally and internally, pushing the latter till the patient was taking every drop that he could bear.

